# From soil to sequence: filling the critical gap in genome-resolved metagenomics is essential to the future of soil microbial ecology

**DOI:** 10.1186/s40793-024-00599-w

**Published:** 2024-08-02

**Authors:** Winston E. Anthony, Steven D. Allison, Caitlin M. Broderick, Luciana Chavez Rodriguez, Alicia Clum, Hugh Cross, Emiley Eloe-Fadrosh, Sarah Evans, Dawson Fairbanks, Rachel Gallery, Júlia Brandão Gontijo, Jennifer Jones, Jason McDermott, Jennifer Pett-Ridge, Sydne Record, Jorge Luiz Mazza Rodrigues, William Rodriguez-Reillo, Katherine L. Shek, Tina Takacs-Vesbach, Jeffrey L. Blanchard

**Affiliations:** 1 Pacific Northwest National Laboratory, Richland, WA 99354 USA; 2 University of California Irvine, Irvine, CA USA; 3 Department of Earth System Science, University of California, Irvine, CA USA; 4 W.K. Kellogg Biological Station, Michigan State University, Hickory Corners, MI USA; 5 Lawrence Berkeley National Laboratory, Berkeley, CA USA; 6 National Ecological Observatory Network - Battelle, Boulder, CO USA; 7 University of California Riverside, Riverside, CA USA; 8 The University of Arizona, Tucson, AZ USA; 9 University of California Davis, Davis, CA USA; 10 Lawrence Livermore National Laboratory, Livermore, CA USA; 11 Life & Environmental Sciences Department, University of California Merced, Merced, CA 95343 USA; 12 University of Maine, Orono, ME USA; 13 Harvard Medical School, Boston, MA USA; 14 University of New Hampshire, Durham, NH USA; 15 University of New Mexico, Albuquerque, NM USA; 16 University of Massachusetts Amherst, Amherst, MA USA

**Keywords:** Soil Microbiome, Hybrid Assembly, Genome Resolved Metagenomics, Microbiome Assembled Genomes, FAIR Data Principles

## Abstract

Soil microbiomes are heterogeneous, complex microbial communities. Metagenomic analysis is generating vast amounts of data, creating immense challenges in sequence assembly and analysis. Although advances in technology have resulted in the ability to easily collect large amounts of sequence data, soil samples containing thousands of unique taxa are often poorly characterized. These challenges reduce the usefulness of genome-resolved metagenomic (GRM) analysis seen in other fields of microbiology, such as the creation of high quality metagenomic assembled genomes and the adoption of genome scale modeling approaches. The absence of these resources restricts the scale of future research, limiting hypothesis generation and the predictive modeling of microbial communities. Creating publicly available databases of soil MAGs, similar to databases produced for other microbiomes, has the potential to transform scientific insights about soil microbiomes without requiring the computational resources and domain expertise for assembly and binning.

## Introduction

Soil microbial communities are incredibly diverse, and they provide crucial services such as supporting plant life, regulating human health [1], and driving global carbon cycling. Soil harbors an immense pool of carbon in the form of roots and decomposed organic matter that exceeds the amount of carbon aboveground in terrestrial plants and the atmosphere combined [2], and in the face of global change it is important to understand how soil microbial communities regulate carbon cycling and other functions. However, the soil microbiome remains largely undescribed; most taxa do not have sequenced genomes, isolation and cultivation of the entire diversity of soil bacteria and archaea is not currently feasible, and, with some exceptions, the viral and eukaryotic components are often not considered [3]. Identifying novel microbial taxa and understanding their influence on microbial diversity and ecosystem-level processes could shed light on methods for mitigating the effects of climate change [4–6].

Shotgun metagenomics has revolutionized our ability to examine complex patterns of functional and taxonomic diversity in soil. Using short read-based sequencing technologies (typically 75–250 base pairs), quality-filtered DNA sequences are used as input to search reference databases for gene function and taxonomy assignments [7]. Taxonomic assignments usually rely on the presence of marker regions, while mapping reads to databases of annotated genes or pathways provides an estimate of metabolic pathway coverage at the community level. Therefore, the estimation of important gene functions and community composition are reliant on separate, independently created and managed databases. A major limitation of this approach is that a scarcity of representative soil bacterial genomes and a lack of robust knowledge of their metabolic capabilities makes it difficult to link community structure with metabolic function.

**Fig. 1:**
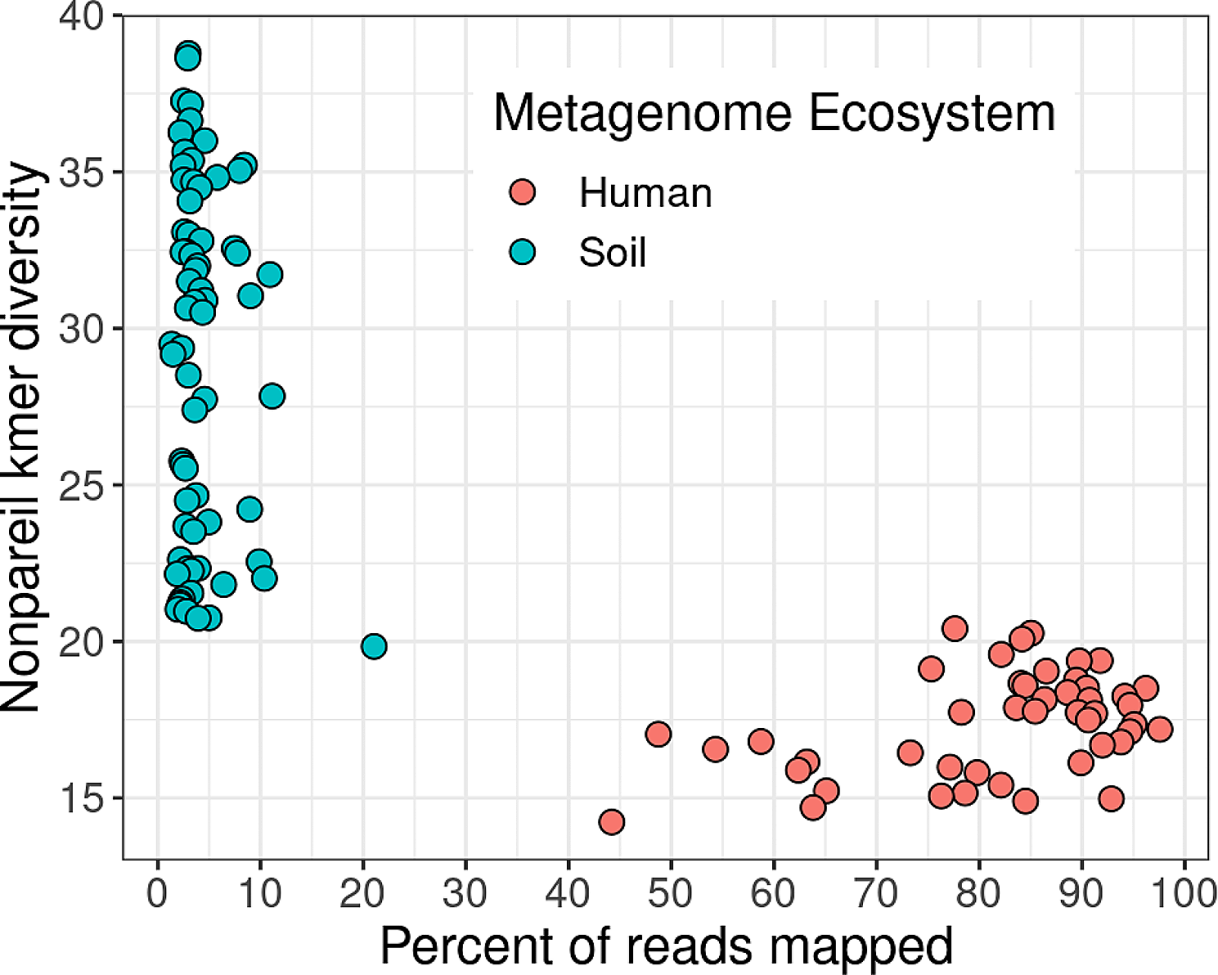
Percent of soil and human metagenomic reads mapped to the GEM and NCBI RefSeq databases as a function of nonpareil kmer diversity. Nonpareil kmer diversity is a measure of genetic diversity within a metagenome. Human microbiomes are less diverse than soil (agricultural, forest and desert) metagenomes. From the efforts of the Human Microbiome Project there is a large collection of bacterial genome sequences in NCBI’s RefSeq database and consequently a large proportion of reads from human metagenomes map to this database. Typically 50–90% of human metagenome reads map to the combined databases. In contrast very few soil metagenomic reads map to genomes in NCBI’s RefSeq, and less than 5% map to the GEM catalog

The Human Microbiome Project (HMP) overcame this limitation on analyzing complex microbiome samples through the compilation of reference genomes and databases, which was a large upfront investment with big impact [8]. This comprehensive catalog of microbiome reference genomes results in the mapping of most human-associated metagenomic reads directly to the HMP genomes stored in Integrated Microbial Genomes & Microbiomes (IMG) from Earth’s Microbiomes (GEM) and NCBI databases [9] (Fig. 1), enabling the development of novel analysis methods and software tools for human microbiome analysis [10, 11].

Alongside the increasing availability of computational resources, improved read assembly into longer contiguous stretches (contigs) has increased the recovery of metagenome assembled genomes (MAGs) [12–14]. MAGs from an individual study can be a subset of the full catalog of larger public MAG databases and isolated genomes. By mapping reads to these MAGs, it is possible to link functional genes to specific organisms, providing a platform to bridge community structure with function [15] and enable finer-scale interrogations of emergent properties such as metabolite sharing and carbon cycling of environmental samples at both organism- and population levels. In this perspective we discuss the current state of GRM in soil microbiome research, cover computational and experimental advances propelling the discovery of microbial genomes from soil samples, and finally propose a roadmap towards an open and accessible database of genome-resolved soil microbiome sequences.

## Genome-resolved metagenomics for soil microbiomes: current state of the field

Largely, the GRM approach has enjoyed greater use within the human microbiome field and other environments, where lower diversity enables better MAG construction and a higher percentage of read mapping. High microbial diversity in soils makes it difficult to resolve complete genomes from metagenomic samples [16], requiring much deeper sequencing and high per-sample costs for retrieving soil MAGs (Fig. 1). However, novel binning (the process of separating metagenomic reads into organism-specific groups) strategies involving sophisticated *c*o- [17] and mixed- [18] assembly and bin refinement [19] strategies have arisen to increase resolving power. The resulting improvement in coverage of individual genomes within the metagenome can increase the number and quality of genome bins. Publicly available and easily accessible MAGs enable reuse by other researchers without requiring the computational resources and domain expertise for assembly and binning.

The recovery of high-quality draft genomes of previously uncharacterized viral and eukaryotic MAGs, though initially understudied, is increasingly an area of interest in soil systems. A recent study described novel giant viruses derived from soil samples from the Harvard Forest LTER site using Fluorescence-activated Cell Sorting (FACS) sorting [20]. Another study curated a database including 726,108 de-replicated viral contigs combining metagenome assembled contigs using prairie soil and public virus databases [3]. A terabase-scale combined assembly from Luquillo Experimental Forest, Puerto Rico, revealed tens of thousands of viruses and tens of partial eukaryotes [21]. These efforts contribute to expanding taxonomic databases such as International Committee on Taxonomy of Viruses (ICTV) [22] and National Center for Biotechnology Information (NCBI) Taxonomy Database [23]. IMG has implemented workflows to analyze viruses and eukaryotes as part of its routine processing. Efforts such as these are critical for building large, increasingly complete databases of soil microbial genomes, expanding our understanding of soil taxa, and facilitating efforts to link genes to specific microorganisms.

Efforts are underway to create environment-specific MAG databases and resources, albeit for less diverse ecosystems. The TARA oceans dataset was leveraged to generate thousands of MAGs from marine environments, while the Genome Resolved Open Watersheds database (GROWdb) focuses on microbes from rivers and streams [24–26]. Resources such as MGnify [27] and organizations such as the National Microbiome Data Collaborative (NMDC) [28] link to or host some of these catalogs, but there are still many environments such as soils that are significantly underrepresented and under sampled. There is a pressing need for high quality soil GRM databases, similar to the Human Microbiome Project that spans soils across space and time building upon collections of cultured soil representatives [29]. A recent step forward involved the reprocessing of soil-related metagenomes into a standalone set of data products including over 40,000 MAGs [30], producing the SMAG catalogue for future use.

## The future of GRM for soil microbiomes

The complexity of soil microbiomes poses several technical hurdles to genome-resolved analyses. Soil is abundant with “relic DNA” [31, 32], defined as extracellular DNA from dead bacterial and fungal cells, which can hide temporal differences in sample from the same community. This can affect estimates of diversity [33]. Metatranscriptomics, which uses the quickly degraded RNA molecule, can be used to assess the functioning members of a microbial community [34]. Though there are DNA intercalation agents which can bind to and remove cell free DNA [31], relic DNA removal and quantification of its effect on analysis is still a developing area of soil microbiome research [32, 33].

Gaps in coverage and repetitive elements in a genome can cause fragmentation in assemblies, leading to incomplete and contaminated sequence bins. The use of long-read sequence platforms such as Oxford Nanopore and Pacific Biosciences, which can span typical microbial repeat lengths of 5–7 Kbp [8], can improve assembly and binning while reducing contamination. These platforms are more error-prone, but are becoming less expensive and more mainstream [35]. Hybrid sequence analysis approaches utilizing long and short reads are now capable of producing higher quality, more contiguous assemblies than either technique alone at lower depth [36]. Tools such as SPAdes (hybridSPAdes) [37] and Unicycler [38] both utilize short reads to produce an initial assembly graph and then close or bridge gaps with contigs assembled from long reads. Multiple studies have attempted to compare the abilities of short, long, and hybrid sequencing approaches for de novo MAG catalog creation [39–43], reporting differences in GC distributions of recovered MAGs, and in the number of detected genes [40]. Most recently, Eisenhofer et al. report that while short reads capture more diversity in recovered MAGs due to higher sequence depth, long read and hybrid assembly strategies result in better assembly statistics. They end their analysis by suggesting that the optimal sequencing/assembly strategy is highly study-specific and will change based on whether MAG quantity or quality is deemed more valuable [43].

Algorithmic advances are improving results for the same input data [44]. For example, machine learning approaches that increase protein function identification are being applied to sequencing data [45, 46] and assembly [47]. Hi-C (capture chromatin conformation) and similar technologies allow for binning based on physical proximity rather than tetranucleotide frequency or sequencing coverage, greatly increasing the ability to identify sequences from the same genome and is used to identify bacteria-phage associations [48, 49]. Finally, there are tools purpose-built to identify contamination and chimerism in prokaryotic genomes, such as checkM [50], and GUNC [51].

An alternative set of approaches aim to reduce complexity through segregation and sorting. Methods such as FACS [52] and Stable-Isotope Probing (SIP) [53] segregate and reduce complex communities, lowering the sequence depth necessary to recover high quality genome bins. Novel viruses identified in samples collected from Harvard Forest LTER were derived from FACS-sorted samples, demonstrating the utility of artificially simplifying complex environments for viral genome enrichment from soil samples [20]. SIP-based methods are traditionally very labor intensive, but recent work has improved throughput [53].

Genome-resolved analyses improve our ability to explore complex soil communities where species’ physiology is unknown and culture-based methods are infeasible. GRM can achieve increased taxonomic resolution for the large diversity of environmental microbes but require robust databases of cumulative genomic knowledge. The fungal component of soil microbiomes is understudied, but recent advances in long read sequencing are opening the field, leading to new insight into fungal diversity and evolution [54]. Efforts to include fungal and eukaryotic microbes in microbiome catalogs are occurring in other environments [55, 56], but soil-specific fungal MAG catalogs are needed. As stated previously, community-driven projects such as TARA, GROWdb, MGnify, NMDC, SMAG, FUNGIDB [57], etc. are excellent initial efforts, and we propose the following goals which should be met or exceeded to build on this foundation:

- Curation: A future soil MAG database needs to contain sampling throughout the entire spectrum of the soil medium. An excellent start is seen in the Joint Genome Institute’s GOLD organism ecosystem classifications, however currently 15,154/23,473 (64.6%) of soil organisms are not classified into a specific ecosystem subtype. A future database requires full FAIR metadata schema compliance [58] and version control for the entirety of its data processing. The creation of a new soils-specific GO FAIR implementation network (https://www.go-fair.org) could work to generate microbiome-specific FAIR practices and tools. We currently recommend following the latest guidance, Minimum Information about any (X) Sequence (MIxS) version 6.2.0, provided by the Genome Standards Consortium (GSC) [59].
- Scale: Initial large scale efforts [8, 60, 61] to survey the human gut microbiome rewarded up-front investment. To recreate those successes in soil microbial ecology, a comprehensive, uniform survey of many different soil types and environments is required, such as the new MONET initiative by the Department of Energy’s Environmental Molecular Sciences Laboratory (EMSL) [62].
- Integration: Ease of use is frequently a barrier to community adoption of new methods and datasets. A future soil MAG database should feature easy integration and data forwarding into KBASE [63], GALAXY [64], The NMDC [28], and other data pipelines and analysis centers. Furthermore, greater acceptance and use of standardized community tools will increase analysis re-producibility. NMDC EDGE (https://microbiomedata.org/workflows/) is a user-friendly web interface community members can use to process their own data in a standardized fashion using community-agreed upon metrics. Tutorials are provided in several languages.

Adoption of these principles in the creation of a MAG database at this scale would require substantial upfront investment. However, in studies where GRM are infeasible due to practical constraints such as cost and sample size, an added benefit of a large MAG database is to make existing and future taxa-based (amplicon) datasets more accurate. Through collaborative efforts to increase the number of high-quality reference MAGs, we can advance our understanding of the biodiversity and ecology of one of Earth’s most complex environments.

## Data Availability

Figure 1 is derived from supplemental materials in Nayfach et al. 2021 [9]. Code to generate the figure is available in the *EMERGENT github repository* https://github.com/lter/lterwg-emergent/tree/master/perspective.
